# Bound exciton and free exciton states in GaSe thin slab

**DOI:** 10.1038/srep33890

**Published:** 2016-09-22

**Authors:** Chengrong Wei, Xi Chen, Dian Li, Huimin Su, Hongtao He, Jun-Feng Dai

**Affiliations:** 1 Department of Physics, South University of Science and Technology of China, Shenzhen 518055, China; 2 Physics Department, The University of Hong Kong, Pokfulam road, Hong Kong, China

## Abstract

The photoluminescence (PL) and absorption experiments have been performed in GaSe slab with incident light polarized perpendicular to c-axis of sample at 10 K. An obvious energy difference of about 34 meV between exciton absorption peak and PL peak (the highest energy peak) is observed. By studying the temperature dependence of PL and absorption spectra, we attribute it to energy difference between free exciton and bound exciton states, where main exciton absorption peak comes from free exciton absorption, and PL peak is attributed to recombination of bound exciton at 10 K. This strong bound exciton effect is stable up to 50 K. Moreover, the temperature dependence of integrated PL intensity and PL lifetime reveals that a non-radiative process, with activation energy extracted as 0.5 meV, dominates PL emission.

The study of two-dimensional crystals has become a very popular topic in the field of condensed matter physics because of its novel phenomena and the potential for optoelectronic and electronic applications. Recently, a new class of 2D crystals, atomically thin metal chalcogenides (such as GaSe^1^, GaTe^2^,^3^ and InSe^4^), has attracted much attention. Take GaSe material as a representative example, it exhibits various interesting physics such as magnetism in p-type monolayer GaSe^5^, non-Markovian memory effects^6^, strong SHE effect in multilayer GaSe^7^,^8^,^9^, optical spin polarization^1^,^10^ and novel behavior of Landau levels in the presence of a Coulombic electron-hole interaction of bulk GaSe^11^,^12^,^13^,^14^,^15^. Actually, the study on GaSe 2D crystal could be traced back to 1958, when the exciton spectrum near fundamental absorption edge of bulk GaSe was first reported^16^. Free and bound exciton behavior in this system has always been discussed^17^,^18^ since its discovery. Here we report the remarkable energy difference between absorption peak and photoluminescence (PL) peak of GaSe slab at low temperature, which is about 34 meV at 10 K. By studying the temperature dependence of PL and absorption spectra, we attribute the absorption peak to free exciton absorption, and the PL peak to bound exciton recombination. So the energy difference of 34 meV origins from energy difference between free exciton and bound exciton states. Meanwhile, the temperature dependence of PL integrated intensity and PL lifetime shows that a non-radiative process contributes most in the PL emission at the temperature above 50 K.

The crystal of GaSe consists of covalently bonded layers by van der Waals stacking, and each layer contains four monoatomic sheets in the sequence of Se-Ga-Ga-Se. Bulk GaSe is generally known as an indirect semiconductor, with the lowest conduction band minimum at M point and the highest valance band maximum at Γ point of the Brillouin zone. However, the energy difference between Γ and M points at the lowest conduction band is in the range of 10–20 meV, hence the direct band gap energy is just slightly greater than the indirect band gap energy. Due to weak interlayer interactions and high electron density within layers, exciton effect is prominent in monolayer and bulk GaSe material. According to Mooser’s theory, optical selection rules near Γ point could be explained by using an exciton (two-particle) picture. When spin-orbit coupling is ignored, orbital symmetries only allow direct transition near Γ point for the case of *E*/*c*, where E is the polarization of light and c is the c-axis of crystal. When spin-orbit interaction is considered, electron transition between the s-like upper most valence band and p-like lowest conduction band near the Γ point is dipole-allowed for the geometry of *E* ⊥ *c*. In this case, the lowest states with total exciton spin S = 1 can be excited. Here we have explored the characteristics of the lowest energy state by absorption and photoluminescence spectra based on the geometry of *E* ⊥ *c*.

Mechanical exfoliated samples from high quality 2H-GaSe single crystals were used in the whole experiments^9^. The GaSe thin slab with length and width of about 1 mm and thickness of about 10*μm* was mounted on sample holder. The photoluminescence and absorption spectra in a transmission geometry were collected with temperature ranging from 10 K to room temperature. During the PL measurement, GaSe slab was normally excited by a CW laser at wavelength of 532 nm. While in the absorption measurement, a supercontinuum white light laser was used. The PL and absorption spectra were obtained by a spectrometer equipped with a cooled charge-coupled device (CCD). The direction of electric field of excited light for both PL and absorption measurement was perpendicular to the c-axis of samples. Besides, ultrafast PL spectra were also employed to study dynamics of electron-hole pairs of GaSe. Samples were excited by a pulsed laser with wavelength of 560 nm, pulse widths of 150 fs and pulse repetition rates of 80 MHz. The time-resolved PL spectra were measured by a single photon avalanche diode with photon timing resolution of 50 ps.

The black curve in Fig. 1a shows the absorption spectrum of a GaSe slab at 10 K. An unambiguous peak (2.12 eV) near the fundamental absorption edge of GaSe is observed under T = 10 K, which can survive even at room temperature. We attribute this sharp absorption peak to an excitonic transition, which was firstly observed in bulk GaSe in 1958 by Fielding Fischer and Mooser^19^. With increase in temperature, the peak position of exciton emission at band edge shifts from 2.12 eV at 10 K to 2.01 eV at 29 K. The temperature dependent energy shift of exciton peak obtained from absorption spectra is shown in Fig. 1b, where black squares are the experimental data and the red line is the theoretical fit. The fitting procedure was performed using Varshni empirical relationship:


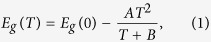


where *E*_*g*_ (0) is the band gap of GaSe slab at 0 K, A and B are constants referred to as Varshni coefficients. The constant A is related to the electron/exciton-phonon interaction and B is related with the Debye temperature of material, which is 251 K for GaSe.

PL spectrum (red curve) from GaSe slab at the same temperature (T = 10 K) is also shown in Fig. 1a. It is observed that the PL spectrum of thin slab consists of multiple lines, where three peaks of PL spectrum are identified at 2.086 eV, 2.064 eV and 2.032 eV respectively (shown in Fig. 1c). In PL spectrum, the highest peak energy is at 2.086 eV, which is 34 meV lower than that of exciton peak at 2.12 eV in absorption spectrum. There is no observable exciton emission in PL spectrum detected around 2.12 eV, which corresponds to the exciton absorption peak in absorption spectrum. In order to rule out the possibility of sample heating by the high-power pump laser applied, which may lead to red shift of PL peak, PL spectrum was also measured under different excitation light intensities. The position of the highest energy peak keeps at around 2.086 eV, independent of excitation light intensity ranging from 30 uw to 600 uw as shown in Fig. 1d. The excessive heat induced by absorption could be completely consumed by cold finger.

This discrepancy between the lowest peak position of PL spectrum and that of absorption spectrum is unusual in intrinsic semiconductor, but it has been frequently observed in the heavily doped semiconductors, where the Fermi level lies within the conduction band and the lowest states in the conduction band are filled^20^. In this case, photon energy larger than the fundamental band gap is needed to excite a transition from valence band to unoccupied states in the conduction band in absorption process. Hence, the absorption peak shifts to higher energy than that of photoluminescence peak as electron-hole recombination occurs near the band edge in photoluminescence process. If this mechanism works, we may deduce the electron density using equation:


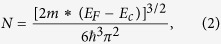


where *E*_*F*_ − *E*_*c*_ is the energy difference between Fermi surface and the bottom of conduction band, and m* is the effective mass near Γ point. Taking *E*_*F*_ − *E*_*c*_ = 34 *meV*, m* = 0.17 *m*_*e*_^21^, and ignoring the spin degeneracy that results in higher electron density, the electron density N is estimated to be 1.96 × 10^18^ *cm*^−3^, 2 orders of magnitude larger than that of intrinsic material. However, electric-conductance measurements showed that GaSe slab is electrically intrinsic. Thus, we may safely rule out this possibility.

This phenomenon was also observed in indirect band gap semiconductor where most absorption occurs in direct transition of electrons around *E*-point under low temperature. After excitation, electrons (holes) are quickly scattered to the lowest point of conduction band near M-point, and then recombine with holes while releasing phonons^22^. This process can result in the red shift of PL spectrum compared with absorption one. Experimental result reported by Mosser’s group^16^ showed that there existed an indirect gap in bulk GaSe, several tens of meV below the direct one and that the indirect gap was only visible in thick sample with light incident along the layer plane. However, our experimental setup with *E* ⊥ *c* configuration is completely different from theirs. Besides, the quantum yield (QY) of our GaSe sample at 10 K is estimated to be 0.05, with a thin film of fluorescent dye as standard with QY of 0.511 given by absolute PL quantum yields measurement system. The measured QY of our GaSe sample is one order of magnitude larger than that of monolayer MoS_2_, which is a direct-bandgap semiconductor^23^. So our GaSe sample is not an indirect gap semiconductor, since the band gap photoluminescence QY for indirect band gap material is usually negligible compared to the direct band gap one for the phonon-assisted process of its photoluminescence. This evidence tends to support the possibility that the PL of our GaSe sample at low temperature is attributed to the direct interband transition. So the hypothesis for its being indirect band gap one is also excluded.

One potential reason may arise from high density of state (DOS) below the top of valance band, which exhibits a sharp van Hove singularity^5^. The DOS of single layer GaSe is almost a step function at the VBM and gives a quickly peak at the energy 0.013 eV below the VBM. Therefore, the energy of strong exciton absorption peak, which emerges at the position of the highest DOS, is higher than that of PL peak resulted from the electron-hole pair recombination near the band gap. However, this unusual character of large DOS at VBM mainly stems from a Mexican-hat-like energy surface around the zone center (Γ) at the monolayer limit^24^. And according to theoretical calculation, GaSe monolayer has an indirect band gap^25^. It is inconsistent with our experimental result that bulk GaSe is a direct band gap semiconductor at low temperature. So this explanation is also excluded.

Figure 2a shows the evolution of PL spectra with increasing temperature under cw laser excitation of 2.33 eV, which is higher than the band gap of GaSe slab. As shown in Fig. 2a, the PL spectrum shifts to lower energy as the temperature increases from 10 K to 40 K. Around 50 K, a higher energy peak around 2.102 eV labeled as “A” emerges, which is absent below 50 K. As temperature goes up to 290 K, the peak A shifts to lower energy, and then merges together with other PL peaks. At room temperature, only one PL peak around 2.00 eV can be observed. We fit the PL spectrum under 60 K with 4 peaks, and label the two highest energy peaks as “A” and “B”. It is shown in Fig. 2b that the energy difference between peaks A and B is around 39 meV at 60 K, which is comparable with the energy difference (34 meV) between high energy PL peak (2.086 eV) and exciton absorption peak (2.12 eV) at 10 K shown in Fig. 1a. Besides, peak A has a FWHM of 10 meV, seven times smaller than that of peak B. Therefore, we could attribute this peak A (around 2.102 eV) emerging at 60 K to the free exciton and peak B at lower energy to the bound exciton. As to peak C and peak D with lower energy than that of peak B, they are probably ascribed to the radiative recombination of trap states^17^. The temperature dependence of this free exciton emission is shown in Fig. 2c, where red spots represent PL peak position of free exciton with FWHMs labeled by red bar, whereas black squares represent exciton absorption peak with FWHMs labeled by black bar at various temperatures. Peak position of this free exciton emission changes from 2.10 eV with a FWHM of 8 meV at 60 K to 2.0 eV with a FWHM of 47 meV at 290 K. And exciton peak of absorption spectrum changes from 2.11 eV with a FWHM of 9 meV at 70 K to 2.01 eV with a FWHM of 14 meW at 290 K. These two peaks almost overlap with each other in the temperature range of 50 K to 290 K. Both of them exhibit the same temperature dependence, which can be fitted by Varshni empirical relationships. All evidences tend to demonstrate that the peak A of PL spectrum stems from radiative recombination of free excitons. Because the probability of thermal dissociation effect in bound excitons increases with the increased temperature, more and more free excitons are released from bound states. Therefore, the PL intensity of free excitons becomes relatively higher than that of bound excitons as temperature rises (Fig. 2a). So under room temperature, most of PL come from the radiative recombination of free excitons. Figure 3c also shows an obvious discrepancy in FWHM of PL and absorption spectra in the temperature range of 150 K–290 K. We only observed a single broad emission peak in PL spectra in this temperature range, which makes it difficult to distinguish free excitons from bound excitons. So the FWHM of PL spectra is rather wider than that of absorption spectra in the range of 150 K to 290 K. Below 50 K, photoexcited free excitons are quickly captured by defect/impurity centers to form bound states, and consequently free exciton is difficult to be detected. These results provide another potential cause for energy difference in PL and absorption spectrum at low temperature.

We also investigated temperature-dependent integrated PL intensity under the same experimental conditions, including excitation power as well as exposure time. As shown in Fig. 3a, the PL intensity dramatically drops by 2 orders of magnitude as temperature rises from 10 K to 50 K, and then has a flat plateau above 60 K. The temperature dependence below 50 K indicates that some non-radiative processes dominate the PL intensity, which are activated by increasing temperature. In such a situation, PL intensity is proportional to the scattering rate of non-radiative process-*I* ∝ exp(−*E*/*k*_*B*_*T*), where E is the activation energy. As shown in Fig. 3b, the black points are integrated intensity at different temperature, and the red curve is a fitting result based on the above equation, from which we extract E = 0.5 meV. Because of the layered structure of GaSe slab, stacking faults and dislocation often occur in the samples. Therefore, non-radiative centers are formed by these defect states, inducing the quenching of PL intensity as temperature rises.

Figure 4a shows lifetime evolution of the main PL peak with temperature. All PL lifetime data were collected around PL peak with bandwidth of 10 nm. Due to limitation of the repetition frequency of the excitation pulse laser, the maximum span of PL lifetime is around 12.5 ns. The black curve in Fig. 4a shows the PL lifetime as a function of delay time at 10 K. Non-zero PL intensity (around 6000 counts) before time zero indicates that PL lifetime at 10 K is longer than 12.5 ns, which is the limitation of our setup. By considering non-zero PL intensity before time zero and fitting PL data by bi-exponential function at 10 K, we deduce two time constants of 1.8 ns and 55 ns as shown in red curve of Fig. 4a. The deduced fast process with time constant of 1.8 ns can be attributed to bound exciton formation, which corresponds to the time that free excitons are captured by defects/impurities. And the slow process of 55 ns is attributed to PL decay of bound exciton. At 260 K, PL decay of free exciton only contains one component with time constant of 2.8 ns. When temperature changes from 10 K to 290 K, PL lifetime (the slow process) sharply decreases from 55 ns at 10 K to 12 ns at 80 K, then PL lifetime approaches to a constant of 2.3 ns above 80 K. The trend of PL lifetime shown in Fig. 4b is consistent with that of PL intensity, which further indicates that non-radiative process dominates at elevated temperature.

In summary, we observe a big energy difference between exciton absorption peak and PL peak below 50 K. By analyzing the temperature dependence of PL and absorption spectra, we propose that the PL mainly origins from emission of bound exciton at low temperature and the absorption peak results from absorption of free exciton at direct gap. By comparing PL intensity and lifetime under various temperatures, the activation energy of non-radiative process is extracted to be around 0.5 meV.

## Additional Information

**How to cite this article**: Wei, C. *et al.* Bound exciton and free exciton states in GaSe thin slab. *Sci. Rep.* **6**, 33890; doi: 10.1038/srep33890 (2016).

## Figures and Tables

**Figure 1:**
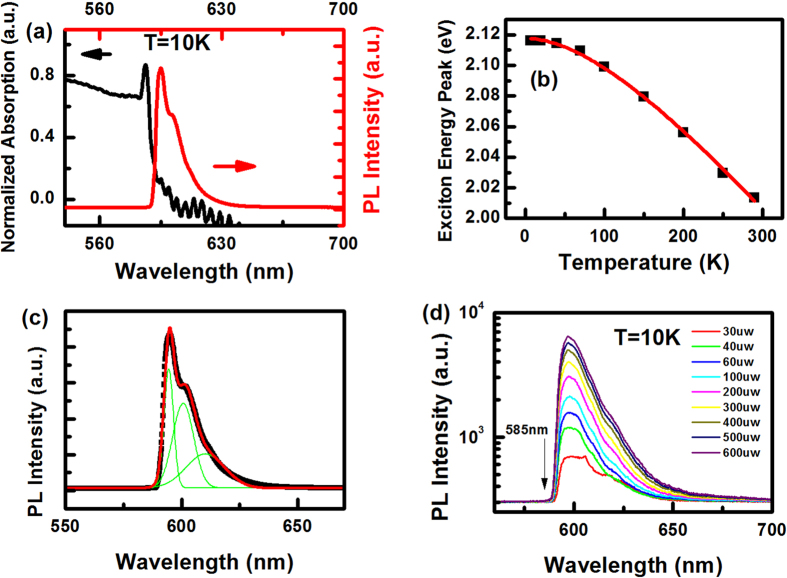
(**a**) Absorption and PL spectrum of GaSe slab at 10 K. (**b**) Exciton energy peak position (black squares) as a function of temperature. The solid red line represents the fitting curve by using Varshni empirical relationship
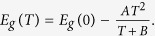
 (**c**) Muli-peak fitting of PL spectrum at 10 K, and energy of these three peaks are identified as 2.086 eV, 2.064 eV and 2.032 eV, respectively. (**d**) PL spectrum as a function of pump intensity ranging from 30 uw to 600 uw at 10 K.

**Figure 2:**
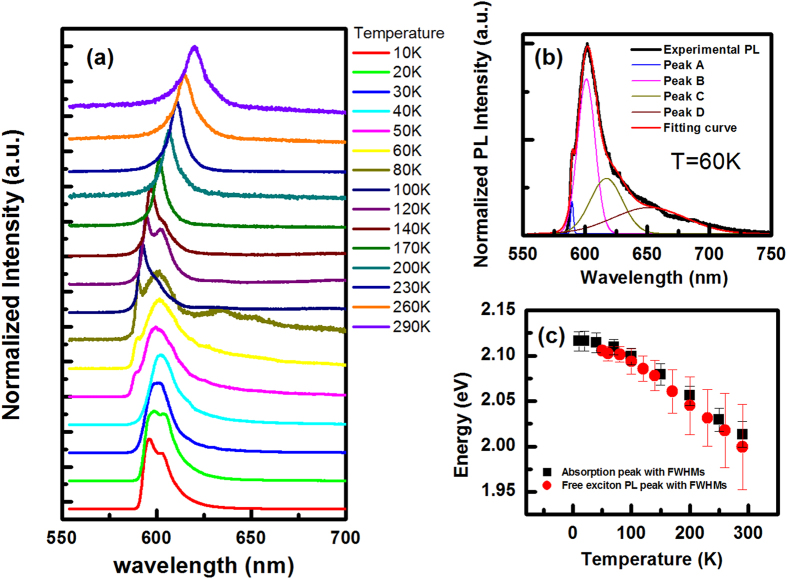
(**a**) Normalized PL spectra under different sample temperature ranging from 10 K to 290 K. (**b**) PL spectrum and muli-peak fitting curves at T = 60 K. (**c**) Absorption peak (black squares) and free exciton PL peak (red circles) as a function of temperature, where bars represent the FWHMs.

**Figure 3:**
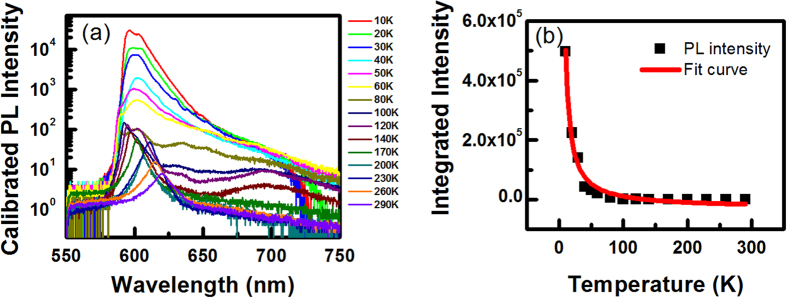
(**a**) PL spectra at various temperatures under the same experimental conditions, including excitation power, CCD optical integral time. (**b**) The integrated PL intensity as a function of temperature (black squares) and fitting curve (solid red curve) by using the equation *I* ∝ exp(−*E*/*k*_*B*_*T*).

**Figure 4:**
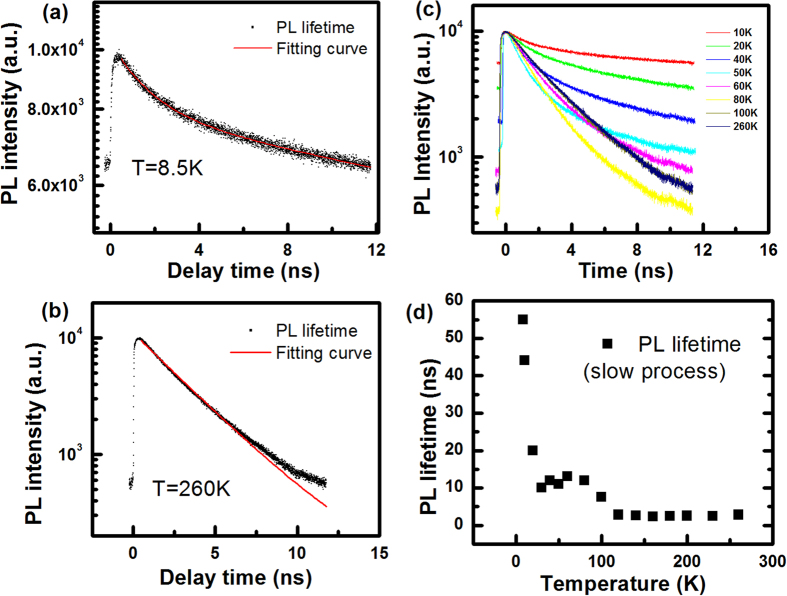
The PL lifetimes as a function of delay time and fitting curve at 8.5 K (**a**) and at 260 K (**b**). (**c**) PL lifetime under different sample temperature. (**d**) The PL lifetime (slow process), extracted from PL intensity vs delay time curve, as a function of temperature.
